# Pre-implantological treatment routines for alveolar ridge atrophy – an investigation among maxillofacial and oral surgeons in southern Germany

**DOI:** 10.1186/s12903-020-01179-3

**Published:** 2020-07-08

**Authors:** Michael Korsch, Winfried Walther, Bernt-Peter Robra, Aynur Sahin, Matthias Hannig, Andreas Bartols

**Affiliations:** 1 Dental Academy for Continuing Professional Development, Karlsruhe, Lorenzstrasse 7, 76135 Karlsruhe, Germany; 2 Clinic of Operative Dentistry, Periodontology and Preventive Dentistry, University Hospital, Saarland University, Building 73, 66421 Homburg, Germany; 3 Center for Implantology and Oral Surgery, 69120 Heidelberg, Germany; 4 Institute of Social Medicine and Health Services Research, Otto-von-Guericke-University of Magdeburg, Magdeburg, Germany; 5 Private Practice, Blumenstrasse 5, 69115 Heidelberg, Germany; 6 School for Dental Medicine, Christian-Albrechts-University Kiel, Clinic for Conservative Dentistry and Periodontology, Kiel, Germany

**Keywords:** Dental implant, Specialists, Oral surgeon, Maxillofacial surgeon, Pre-implantological treatment, Bone augmentation, Bone resection

## Abstract

**Background:**

It is not well-known which pre-implantological procedures are preferred by maxillofacial (MFS) and oral surgeons (OS) for the narrow atrophic alveolar ridge under practice based conditions and, if different training paths in surgery lead to other pre-implantological techniques being preferred. This study aims to identify which procedures are preferred by the respective specialists in which indication.

**Methods:**

A questionnaire was sent to a total of 300 MFS and OS in southern Germany. The questionnaire examined pre-implantological procedures (bone block, bone grafting material and/or particulate autogenous bone, titanium mesh, bone split, resection) in the edentulous severely atrophic mandible and in the severely atrophic single-tooth gap. Kendall’s Tau-b test was used for statistical analyses.

**Results:**

One hundred seventeen participants returned the questionnaire. 68 (58%) were OS and 49 (42%) were MFS. In the edentulous mandible, bone substitute material and resection were most preferred by both specialists. Bone blocks were statistically significantly more frequently associated with MFS and bone substitute materials with OS. Bone split was more frequently used in the atrophic single tooth gap than in the edentulous mandible. OS preferred bone blocks in the single tooth gap more often than in the edentulous mandible. MFS and OS preferred resection in the edentulous mandible significantly more frequently than in the single tooth gap.

**Conclusions:**

MFS in general prefer more invasive pre-implantological therapies with the same initial diagnosis than OS, which seems to be attributed to different training paths.

## Background

With progressive resorption of the alveolar ridge, the conditions for implant surgery become less favourable. Here, bone loss can occur in horizontal or vertical dimensions or combined in horizontal and vertical dimensions and in the upper jaw as a bone deficit at the border of the maxillary sinus. In such cases, an inadequate implant site can complicate or even impede optimal implant positioning. This in turn can have an influence on the prosthetic treatment result, because the optimal design of the prosthetic superstructure is decisively determined by the correct implant position [1]. In cases with advanced resorption of the alveolar ridge, pre-implantological procedures are therefore frequently required that enable successful implant-supported rehabilitation [2]. The current state of the art in surgical technique allows a functionally and aesthetically satisfactory result to be achieved with appropriate augmentation methods, even in cases where the bone width is primarily insufficient [3, 4]. Afterwards a functional replacement of missing teeth is possible by implant-supported dentures.

A sufficient bone volume for a complete bony coverage of the implant at the time of implantation is crucial to achieve successful long-term results. Therefore, the correct selection of the appropriate pre-implantological technique is important [5]. There are various pre-implantological methods that allow implant-supported dentures to be used in patients with an atrophic alveolar ridge. Additive, expansive and subtractive methods that differ greatly in effort and invasiveness can potentially be used. The aim of all methods is to achieve a sufficiently broad bone width and/or bone height to place an implant. However, there is no generally applicable rule that assigns a technique to be favoured to specific clinical situations. The available treatment methods for the narrow atrophic alveolar ridge differ with regard to the financial and time expenditure, the degree of difficulty as well as the intra- and postoperative burden on the patient.

In the course of his or her professional life, every physician develops clinical routines which she or he uses in practice to provide good and reliable care [6]. These routines include diagnostics and decision making as well as the execution of the actual medical intervention. However, the selection of a specific implantological technique seems to depend on the educational background and the educational institution of the physician rather than on the clinical findings of the case [7]. In Germany, implantological and augmentative procedures are allowed to be performed by any dentist. However, maxillofacial surgeons as well as oral surgeons receive profound implantological training as part of their specialist qualification. Maxillofacial surgeons study both human medicine and dentistry. Afterwards, a five-year specialist training takes place in an authorized institution. The requirement for the specialist training to become an oral surgeon is the successfully completed study of dentistry. Afterwards, a four-year specialist training takes place in a specialist centre under the supervision of an authorised oral surgeon or maxillofacial surgeon.

It is not yet known which pre-implantological procedures are preferred by surgeons for the narrow atrophic alveolar ridge under practice based conditions. In addition, it is unclear whether different training paths in maxillofacial surgeons and oral surgeons lead to other pre-implantological techniques being preferred in atrophied alveolar ridges and which predictors influence their decision. Therefore, the present study aims to identify which pre-implantological procedures are preferred by the respective clinical specialists in which clinical indication and why. Here, this research focusses on the management of horizontal defects of tooth gaps in the mandible and on the management of horizontal defects in the edentulous mandible.

## Methods

A questionnaire was prepared for the present study and sent by postal mail to all resident maxillofacial surgeons as well as oral surgeons in the southern German states of Baden-Württemberg, Bavaria, Hesse, Rhineland-Palatinate and Saarland. Together with the State Dental Chambers of the respective federal states, a total of 300 practicing specialists for maxillofacial surgery or oral surgery were identified, who were also authorized to conduct specialist training in oral surgery. The authorization for specialist training was intended to ensure that the surgeons involved in the study had considerable practical clinical experience and a large number of cases of surgical interventions, which is a prerequisite for getting the authorization to train specialists to be. Before the questionnaire was sent out, an attempt was made to contact all potential study participants by telephone to explain the aim of the survey and thus increase the response rate as much as possible. If a telephone contact could not be established, the questionnaire was sent without further notice anyway.

Excluded from this study were dentists or physicians who did not have the specialist designation for maxillofacial surgery or oral surgery, surgeons without authorisation for specialist training and surgeons who worked at hospitals.

The present study was reviewed and approved by the Ethics Committee of the Saarland Medical Association (Ref. No.: 133/11).

The questionnaire examined possible pre-implantological procedures in the case of the edentulous severely atrophic mandible and in the case of the severely atrophic single-tooth gap.

The questionnaire consisted of several parts. In the first part, physician- and practice-related characteristics were collected. In the second part, the clinical routines for pre-implantological procedures on the severely atrophied alveolar ridge were assessed. The questionnaire can be found in the supplementary files (S1). Before the questionnaire was sent out, it was tested for comprehensibility and practicability by a convenient sample of five surgeons known to the study authors. These surgeons were not part of the study sample and were blinded.

The following physician- and practice-related characteristics were collected: Referral practice, individual or shared practice/joint practice; specialist for maxillofacial surgery or specialist for oral surgery; number of professional years after specialist training; postgraduate continuing education: Completion of a Master of Science (MSc) or implantology curriculum; number of implants placed in the last year (self-assessment); number of edentulous mandibles treated with implants in the last year (self-assessment); availability of a cone beam computed tomography system (CBCT) for three-dimensional imaging of the maxillofacial bone.

In the next section, the various pre-implantological procedures for the atrophic alveolar ridge either in the interforaminal area of the edentulous mandible or in the single tooth gap were assessed (for description of the various procedures see Table 1). This section included additive (bone block, augmentation with bone grafting material and/or particulate autogenous bone, titanium mesh), expansive (bone split) and subtractive (resection) pre-implantological methods as therapy options. Distraction was used as a negative blank feed for the survey. The answers to the individual questions were given on a Likert-type scale from 0 to 5 (0 = not used by me, 5 = used very frequently by me). In addition, the frequency at which the specialists prefer which donor region in the case of bone harvesting for alveolar ridge augmentation was determined.

**Table 1:** Description of the different pre-implantological procedures for the atrophied edentulous mandible and the atrophied single tooth gap surveyed in the study

Procedure	Description of the respective procedure
*Bone split*	Splitting the alveolar ridge and mobilization of the buccal and oral bone lamella.
*Bone block*	Transplantation of a bone segment and fixation with screws.
*Bone substitute material*	Augmentation with bone substitute material and/or particulate bone (without further procedures).
*Distraction*	Mobilisation of a bone segment formed by separation of the jaw by means of a distraction device.
*Mesh*	Jaw augmentation with bone substitute material and/or particulate bone held by a titanium mesh.
*Resection*	Removal of pointed alveolar residual bone to achieve a sufficiently wide implant site.

In the last section, the frequency of special diagnostic procedures such as model analysis, CBCT diagnostics and construction of a drilling template with or without computer-aided 3D planning were surveyed.

The last question was which type of prosthetic suprastructure (fixed/removable) is preferred in the atrophied edentulous mandible or whether the prosthetic restoration is decided and performed by the referring dentist.

The data from the questionnaires were collected with Microsoft Excel and evaluated with IBM SPSS Statistics 22 (IBM SPSS Statistics, IBM, Armonk, New York, United States) operating on Microsoft Windows 7. The evaluation was performed with complete data sets. Missing data of the study participants were not considered. The Chi^2^ test was used as the statistical method for binary data, mean value comparisons were carried out using ANOVA and rank scale comparisons with the non-parametric Kendall’s Tau-b test. A statistically significant difference was assumed at *p* < 0.05.

## Results

A total of 300 resident surgeons were identified of whom 145 were oral surgeons and 155 were maxillofacial surgeons. Fifty of these surgeons (OS *N* = 23, MFS *N* = 27) refused to participate in the telephone contact. The reasons for non-participation were: no interest in study (*N* = 23), no time (*N* = 20), retired/not working any more (*N* = 4), long-term sick (*N* = 1), does not place implants (*N* = 1) and wrong adress (*N* = 1). The questionnaire was sent to the 250 remaining surgeons and answered by 117 colleagues. The response rate was 39.0%. Of the 117 participants who answered the questionnaire, 49 (41.9%) were specialists in maxillofacial surgery, and 68 (58.1%) were specialists in oral surgery. Twenty six (53.1%) of the 49 specialists in maxillofacial surgery had the additional designation of oral surgery.

### General physician- and practice-related characteristics

Thirteen (11%) of the 117 study participants were female. However, there were no significant differences in gender distribution between the group of maxillofacial surgeons and oral surgeons (Chi^2^ = 0.742, *p* = 0.39). Fifty-nine percent of the interviewees were active in joint practices and 41% in individual private practices. Maxillofacial surgeons were significantly (Chi^2^ = 12.494, *p* < 0.001) more frequently active in referral practices (80%) than oral surgeons (47%). The average professional experience of the participants after completion of specialist training was 18.7 (SD 7.5) years. There were no statistically significant differences between maxillofacial and oral surgeons (ANOVA F = 0.099, *p* = 0.75). Sixty-four percent of all surgeons had completed a curriculum for implantology and 13% of the respondents had the additional title Master of Science (MSc). The number of practices equipped with CBCT technology were not significantly different between maxillofacial and oral surgeons (Chi^2^ = 1.711, *p* = 0.19). However, maxillofacial surgeons placed a significantly higher number of implants per year (Chi^2^ 12.185, *p* = 0.007) and treated a significantly higher number of edentulous mandibles per year using implants (Chi^2^ 12.703, *p* = 0.005) compared to oral surgeons. The detailed physician- and practice-related characteristics of the study participants are summarized in Table 2. The analysis with regard to the distribution of specialist training between the participant groups and the non-responder groups did not reveal any statistically significant differences.

**Table 2:** Detailed physician- and practice-related characteristics of the study participants

		Study group		Significance between Groups OS and MFS
Baseline data of participants	Total	OS	MFS	*p*-Value
**Total returned questionnaires*n*(%)**	**117 (100)**	68 (58)	49 (42)	Chi^2^ = 3.085, *p* = 0.08
**Gender (male)**				
***n*(%)** related to total returned questionnaires	**104 (89)**	59 (87)	45 (92)	Chi^2^ = 0.742, *p* = 0.39
**Years of experience after specialist designation**				
**Mean (SD)**	**18.7 (7.5)**	18.5 (8.0)	18.9 (7.0)	ANOVA F = 0.099, *p* = 0.75
**Pure referral practice (answered by (%))**	**113 (100)**	64 (57)	49 (43)	
Yes*n*(%)^*^	**69 (61)**	30 (47)	39 (80)	Chi^2^ = 12.494, ***p*** **< 0.01**
**Joint practice (answered by (%))**	**115 (100)**	66 (57)	49 (43)	
Yes*n*(%)^*^	**56 (49)**	30 (46)	26 (53)	Chi^2^ = 0.651, *p* = 0.42
**Shared practice** (e.g. only shared rooms, equipment) **(answered by (%))**	**115 (100)**	66 (57)	49 (43)	
Yes*n*(%)^*^	**12 (10)**	7 (58)	5 (42)	Chi^2^ = 0.005, *p* = 0.94
**Private single practice (answered by (%))**	**115 (100)**	66 (57)	49 (43)	
Yes*n*(%)^*^	**47 (41)**	29 (44)	18 (37)	Chi^2^ = 0.604, *p* = 0.44
**Completed curriculum for implantology (answered by (%))**	**110 (100)**	62 (56)	48 (44)	
Yes*n*(%)^*^	**70 (64)**	40 (65)	30 (63)	Chi^2^ = 0.048, *p* = 0.83
**Master of Science (MSc) in implantology (answered by (%))**	**111 (100)**	63 (57)	48 (43)	
Yes*n*(%)^*^	**14 (13)**	8 (13)	6 (13)	Chi^2^ = 0.001, *p* = 0.98
**No of implants placed in the last year (own estimate) (answered by (%))**	**110 (100)**	62 (56)	48 (44)	
0–100*n*(%)^*^	**10 (9)**	10 (16)	0 (0)	
100–500*n*(%)^*^	**61 (56)**	36 (58)	25 (52)	
500–1000*n*(%)^*^	**32 (29)**	14 (23)	18 (38)	
> 1000*n*(%)^*^	**7 (6)**	2 (3)	5 (10)	Chi^2^ = 12.185, ***p*** **< 0.01**
**No of edentulous mandibles treated last year (answered by (%))**	**110 (100)**	62 (56)	48 (44)	
0–10*n*(%)^*^	**19 (17)**	17 (27)	2 (4)	
10–20*n*(%)^*^	**40 (36)**	23 (37)	17 (35)	
20–50*n*(%)^*^	**27 (25)**	13 (21)	14 (29)	
> 50*n*(%)^*^	**24 (22)**	9 (15)	15 (31)	Chi^2^ = 12.703, ***p*** **< 0.01**
**CBCT/CT equipment in practice (answered by (%))**	**110 (100)**	62 (56)	48 (44)	
Yes*n*(%)^*^	**61 (56)**	31 (50)	30 (62)	Chi^2^ = 1.711, *p* = 0.19

*OS* Oral surgeons, *MFS* Maxillofacial surgeons, bold *p*-Values indicate a statistically significant difference with *p* < 0.05, ^*^ – Percentages in respect to the totals of the corresponding section

### Pre-implantological procedures for the preparation of an appropriate implant site in the edentulous mandible

**Fig. 1:**
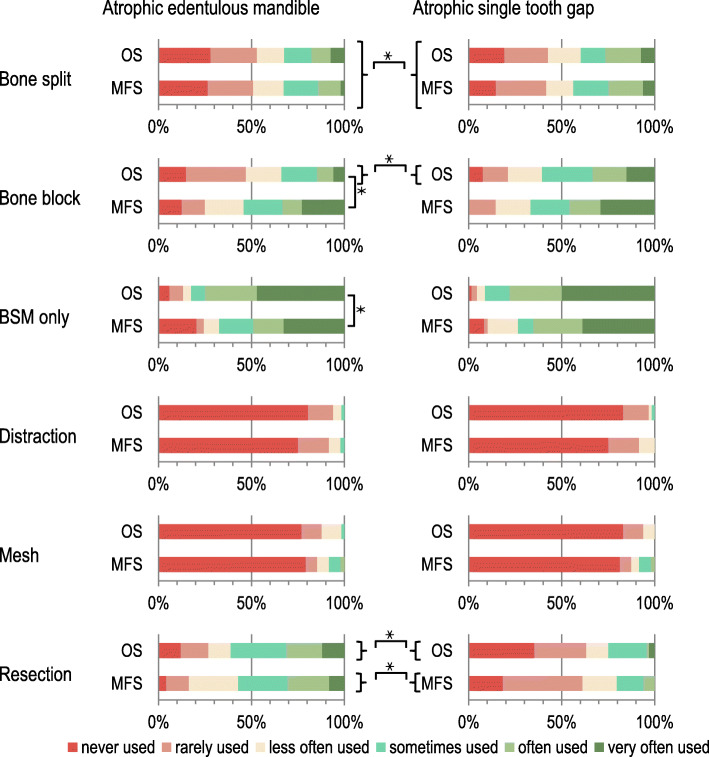
Preferred pre-implantological procedures of maxillofacial and oral surgeons for implant site preparation in the atrophic edentulous mandible or in the atrophic single tooth gap. Legend: OS – oral surgeons, MFS – maxillofacial surgeons, * - shows statistically significant differences between OS and MFS or between the two indications of atrophic edentulous mandible and atrophic single tooth gap (Kendall’s τ_b_; *p* < 0.05)

In the edentulous mandible, the following pre-imlantological procedures were preferred by both groups of specialists in decreasing frequency for the preparation of a suitable implant site (Fig. 1): bone substitute material, resection of the alveolar ridge, bone block, bone split, titanium mesh and distraction techniques. Titanium mesh and distraction techniques were virtually not used (86.7% (mesh) and 93.0% (distraction) mentions ‘rare’ or ‘never’ in total combined for both specialist groups). Sonic Weld (three times listed) [8], diameter-reduced implants (two times listed), the use of plasma rich growth factor (once listed) [9], the Khoury bone block shell technique (once listed) [10] and segment osteotomy with V-Y plate (once listed) were mentioned as “other” types of therapy in both groups.

The use of bone blocks was statistically significant more frequently associated with the specialist designation ‘maxillofacial surgeon’ (τb = − 0.215, *p* = 0.009) and the use of bone substitute materials was statistically significantly more frequently associated with the specialist designation ‘oral surgeon’ (τb = − 0.169, *p* = 0.047) (see Fig. 1).

### Pre-implantological procedures for the preparation of an appropriate implant site in the single tooth gap

In the case of the single tooth gap, the following pre-imlantological procedures for the preparation of a suitable implant bed were preferred by both groups of specialists in decreasing frequency (Fig. 1): bone substitute material, bone block, bone split, resection of the alveolar ridge, titanium mesh and distraction techniques. Titanium mesh and distraction techniques were nearly never used in this indication (91.2% (mesh) and 94.7% (distraction) mentions ‘rare’ or ‘never’ in total combined for both groups). None of all pre-implantological procedures surveyed was statistically significant associated with one of the specialist groups.

The combined group of surgeons (MFS and OS) with less than 20 years of professional experience used bone block grafts in the single tooth gap significantly more frequently than surgeons with more than 20 years of professional experience (τb = − 0.200, *p* = 0.012). Surgeons (MFS and OS combined) with a master’s degree used the mesh technique significantly more frequently than surgeons without a master’s degree (τb = 0.220, *p* = 0.020) in the single tooth gap. If a CBCT was available in practice, the mesh technique (τb = 0.205, *p* = 0.031) was used significantly more frequently in the single tooth gap. Surgeons (MFS and OS combined) placing more than 500 implants per year used bone blocks significantly more frequently (τb = 0.239, *p* = 0.006; τb = 0.304, *p* = 0.001) in the single tooth gap than surgeons placing fewer implants.

### Differences between the pre-implantolocial procedures for the edentulous mandible and the single tooth gap

The group of all surgeons (OS and MFS combined) preferred the bone split in the indication of the atrophied single-tooth gap significantly more often than in the edentulous mandible (τb = 0.118, *p* = 0.041). Furthermore, the total group of all surgeons (OS and MFS combined) preferred resection in the edentulous mandible statistically significantly more often than in the single tooth gap (τb = − 0.389, *p* < 0.001). Also within both respective specialist groups, resection in the edentulous mandible was statistically significantly preferred to the single tooth gap (OS: τb = − 0.425, *p* < 0.001; MFS: τb = − 0.373, *p* < 0.001). The bone block was preferred by oral surgeons significantly more often in the indication of a single-tooth gap compared to the edentulous mandible (τb = 0.262, *p* < 0.001). In the group of maxillofacial surgeons there were no statistically significant differences between the two indications for the use of the bone block (τb = 0.140, *p* < 0.116) (Fig. 1).

With regard to physician-related characteristics, surgeons (OS and MFS combined) who completed a 'curriculum implantology' were statistically significantly less likely to perform pre-implantological resection measures (τb = − 0.169, *p* = 0.047) in the edentulous mandible than surgeons without such a curriculum. Other physician- and practice-related characteristics did not show any associations to the various pre-implantological procedures in the edentulous mandible.

### Donor regions for augmentative pre-implantological treatment

**Fig. 2:**
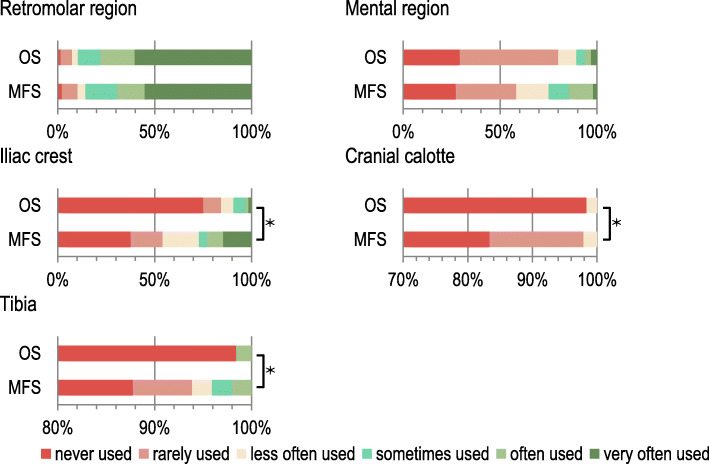
Preferred intraoral and extraoral donor regions of maxillofacial and oral surgeons for pre-implantological implant site preparation. Legend: OS – oral surgeons, MFS – maxillofacial surgeons, * - shows statistically significant differences between OS and MFS (Kendall’s τ_b_; *p* < 0.05)

The following donor regions were named by both specialist groups in descending order of frequency: Retromolar region, mental region, iliac crest, tibia and cranial calotte. Tibia and cranial calotte were very rarely used procedures. The iliac crest, tibia and cranial calotte were statistically significant more frequently associated with maxillofacial surgeons (τb = 0.362, *p* < 0.001; τb = 0.266, *p* < 0.005 and τb =0.213, *p* = 0.024 respectively) compared to oral surgeons (Fig. 2).

### Pre-implantological diagnostics and planning

**Fig. 3:**
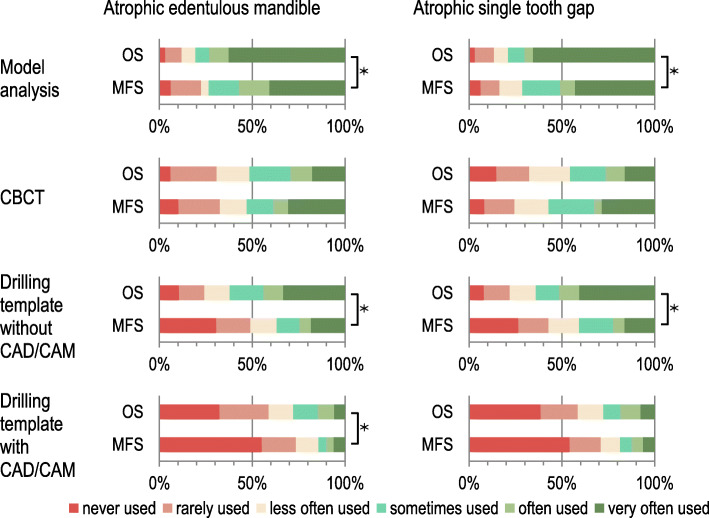
Preferred pre-implantological diagnostics of maxillofacial and oral surgeons for implant site preparation in the atrophic mandible or in the atrophic single tooth gap. Legend: OS – oral surgeons, MFS – maxillofacial surgeons, * - shows statistically significant differences between OS and MFS or between the two indications of atrophic edentulous mandible and atrophic single tooth gap (Kendall’s τ_b_; *p* < 0.05)

In the edentulous mandible, the use of model analysis and surgical drilling guides with and without 3D planning was statistically more often associated with the group of oral surgeons (τb = − 0.189, *p* = 0.028; τb = − 0.193, *p* = 0.022 and τb = − 0.247, *p* = 0.003) compared to maxillofacial surgeons. Model analysis and the surgical drilling guide without 3D planning were also used significantly more frequently by oral surgeons than by maxillofacial surgeons in the single-tooth gap (τb = − 0.181, *p* = 0.035 and τb = − 0.277, *p* < 0.001) (Fig. 3).

### Prosthetic suprastructure in the edentulous mandible

In the group of maxillofacial surgeons, the prosthetic suprastructure was made significantly more frequently by referrers than in the group of oral surgeons (τb =0.293, *p* < 0.001). In general (both specialist groups combined), edentulous mandibles were statistically significant more frequently treated with removable than fixed restorations (τb = − 0.271, *p* < 0.001).

## Discussion

In the present questionnaire study we were able to obtain the expertise of more than one-third of the maxillofacial and oral surgeons in the southwestern region of Germany in order to determine their preferred pre-implantological treatment methods in case of the narrow atrophied alveolar ridge in the edentulous mandible and in case of the atrophic single-tooth gap. As therapy options, additive (bone block, augmentation with bone grafting material and/or particle autogenous bone, titanium mesh), expansive (bone split) and subtractive (resection) pre-implantological treatment options could be selected in order to create a sufficient implant site. The evaluation showed that some techniques to achieve a sufficient implant site were associated both with the indication as well as with the specialist designation.

In the edentulous mandible, bone substitute material, resection of the alveolar ridge, bone block and bone split were the predominant techniques. Bone substitute material and resection were methods that were used particularly frequently in the various indications. In the case of the single-tooth gap, bone substitute material, bone block and bone split were the predominant techniques. Compared to the edentulous mandible, resection had only little relevance in the treatment of the single-tooth gap.

Both, in the edentulous mandible and in the single-tooth gap, the use of bone substitute material and/or particulate bone was most frequently mentioned. The reason for this is probably the lower invasiveness [11] as well as the lower complexity of this therapy. However, there were clear differences in the preference of the other techniques for widening the implant site. While resection in the edentulous mandible was frequently used, this technique was rarely used for the single tooth gap. One explanation for this could be that edentulous patients are usually older than patients with single-tooth gaps. The clinician probably expected that the use of bone substitute material or the resection technique will shorten the treatment time for the patient. With both techniques, the placement of implants can usually take place simultaneously with the pre-implantological procedure. Due to the higher age of patients, complex and time-consuming treatments, such as two-phase interventions, were probably intuitively avoided by the surgeons. Antoun et al. [12] were able to show that significantly higher complication rates occur with edentulous mandibles in two-phase procedures, which is usually the case with titanium mesh, bone block and distraction. Consequently, older patients should be offered therapies with less postoperative discomfort and shorter treatment times. Apparently, the study participants surveyed, predominantly followed this strategy.

Although resection has the disadvantage that bone height is lost through this therapy option, however, this does not seem to have any influence on decision making in the indication of the edentulous mandible. The much rarer choice of resection as a form of therapy in the single-tooth gap probably relates to prosthetic planning. While fixed suprastructures are usually planned in the single tooth gap, the design of which is largely based on the adjacent teeth, the restoration of the edentulous jaw can be designed independently from the surrounding structures. The loss in height caused by resection in the edentulous mandible can be compensated for a large extent without functional and aesthetic restrictions by the subsequent dental prosthesis. In the case of the single-tooth gap, however, resection leads to different bone levels between adjacent teeth and the implant region. This involves the risk of increased periodontal pocket depths and can, therefore, promote peri-implantitis [13].

The bone split technique was mentioned significantly less frequently in the case of the edentulous alveolar ridge than in case of the single-tooth gap. Most surgeons probably decided in favour of resection in the case of the edentulous jaw because simultaneous implantation is normally possible. In contrast, simultaneous implant placement is not always possible with the bone split [14]. Moreover, resection of the alveolar ridge is less technique sensitive than the bone split technique. Additionally, if the wound margins are to be tension-free covered with this technique, mobilisation of the mucoperiosteal flap is usually required. However, this often leads to an increased swelling due to the soft tissue trauma [15]. Overall, the bone split technique is therefore more often subject to complications than the resection technique.

Within the scope of this study it was not possible to clarify the question whether the type of the planned implant supported superstructure had an influence on the selection of the preferred pre-implantological treatment option. This is a limitation of the study. Particularly in the edentulous mandible, different prosthetic restoration methods are possible. A patient who needs two implants to support his denture will obviously benefit much more from a bone resection than from an augmentation especially in terms of treatment costs, morbidity, time and invasiveness amongst others. On the other hand, augmentation can be more advantageous to our practical experience in terms of a better oral hygiene in a fixed full-arch rehabilitation of the edentulous jaw with 6–8 implants. To the authors’ knowledge, there are currently no studies available that investigate whether the planned superstructure will influence the choice of pre-implantological treatment methods.

In addition to the indication, surgical training also had an influence on the choice of therapy. We found that different pre-implantological procedures were more likely to be associated with maxillofacial surgeons or oral surgeons. Maxillofacial surgeons performed more invasive procedures such as bone block transplants in the single-tooth gap and bone harvesting at the iliac crest in contrast to oral surgeons. Oral surgeons significantly more often chose the less invasive procedure of augmentation with bone grafting materials in the single-tooth gap than maxillofacial surgeons. The significantly more frequent use of iliac crest transplants by maxillofacial surgeons can be explained by the different training, since maxillofacial surgeons also learn and perform extraoral surgery while oral surgeons mainly learn intraoral procedures during their training. There is evidence in the literature that training and experience can have a significant influence on the therapy decision and its implementation [16–18]. Furthermore, it is remarkable that some surgeons, apparently due to their training, still practice methods such as iliac crest harvesting, although it has been shown that the resorption rate of these grafts is at best comparable but not lower than with alternative methods [19]. However, the loss rate of iliac crest grafts is higher compared to grafts harvested from the mandible [19].

There were also significant differences in pre-implantological planning methods between the two groups of specialists. Maxillofacial surgeons rarely used model analyses and drilling templates, while oral surgeons used drilling templates more often. In addition, the prosthetic restoration of the placed implants was more frequently performed by oral surgeons themselves, whereas in the case of maxillofacial surgeons the referring physicians usually performed the prosthetic treatment. One reason for this finding could be that oral surgeons are less frequently active in referral-only practices than maxillofacial surgeons and therefore have their own patient base, which they treat completely in dentistry.

A general problem in evaluating the various pre-implantological techniques discussed in this publication is that a clear evidence-based hierarchy of all options is currently difficult. Although different augmentation techniques have often been compared in the scientific literature [3, 20–23], to the authors’ knowledge there is e.g. no study comparing augmentation techniques with the resection of jaw bone to produce a suitable implant site. Furthermore, it seems uncertain whether augmentation methods using bone replacement material only or autologous bone only are superior to the other material. A review on this question concluded that bone substitute material might be as effective for augmentation as autologous bone [24] regarding the survival of implants placed in the respective grafts when used for horizontal augmentation. In another review it was found that autologous bone, allogenic bone and xenografts only were associated with lower bone gain than a mixture of autologous bone and xenograft for horizontal augmentation [25]. In addition, it is not clear whether the differences in the preference for different methods between maxillofacial and oral surgeons are due to the fact that other patient groups with different objective treatment needs and varying degrees of difficulty may visit the corresponding specialist group from the outset and then be treated individually.

In the present study the response rate was 39% of the originally identified oral and maxillofacial surgeons. This seems to be a quite low response rate and is a limitation of this study. However, the response rate must be considered in the light of which rates can be expected for questionnaire studies of a similar type. In our study no incentives were provided. In this case a lower response rate must generally be expected. Mehlkop and Becker [26] stated that a response rate of approximately 28% was to be expected if the respondents did not receive any reward and of approx. 52% if an incentive was provided. Accordingly, we still achieved quite a good response rate in the present study.

## Conclusions

To the authors’ knowledge, this study is the first survey among German oral surgeons and maxillofacial surgeons to examine which pre-implantological measures and techniques and which pre-implantological diagnostics the two specialist groups working in the oral and maxillofacial region actually apply in their practice. The results of our study indicate that maxillofacial surgeons prefer more invasive pre-implantological therapies with the same initial diagnosis than oral surgeons which we mainly attribute to different training paths.

## Supplementary information

**Additional file 1.** S1 Study questionnaire 'Determinants of pre-implantological augmentation procedures'

## Data Availability

Datasets of the current study are available from the corresponding author on reasonable request.

## Abbreviations

CBCT: Cone beam computed tomography
OS: Oral surgeon
MFS: Maxillofacial surgeon
MSc: Master of Science
No.: Number
Ref. No.: Reference number
