# Supplementary material for: Pre-implantological treatment routines for alveolar ridge atrophy – an investigation among maxillofacial and oral surgeons in southern Germany
Source: BMC Oral Health. 2020 Jul 8;20:195. doi: 10.1186/s12903-020-01179-3 (PMC7346617; doi:10.1186/s12903-020-01179-3)
Supplement: Supplementary file 1 — Additional file 1. S1 Study questionnaire 'Determinants of pre-implantological augmentation procedures' [file 12903_2020_1179_MOESM1_ESM.docx]

**Determinants of pre-implantological augmentation procedures – a questionnaire study**

| 1. **Practice profile**   Please indicate which type of practice you have and which qualifications you hold. | | Yes | No |
| --- | --- | --- | --- |
| 1.1 | Do you run a pure referral practice? | 🞏 | 🞏 |
| 1.2 | Do you work in a joint practice? | 🞏 | 🞏 |
| 1.3 | Do you work in a shared practice (e.g. only shared rooms and equipment)? | 🞏 | 🞏 |
| 1.4 | Do you work in a private single practice? | 🞏 | 🞏 |
| 1.5 | Are you a specialist in maxillofacial surgery? | 🞏 | 🞏 |
| 1.6 | Are you a specialist for oral surgery? | 🞏 | 🞏 |
| 1.7 | For how many years have you had a specialist designation in maxillofacial surgery or a specialist designation in oral surgery? | ______ years | |
| 1.8 | Have you completed a curriculum for implantology? | 🞏 | 🞏 |
| 1.9 | Have you completed a Master of Science (MSc) degree programme? | 🞏 | 🞏 |

| 1. **Augmentation techniques**   Which augmentation techniques do you use? We ask you about the techniques that are most commonly used by you for the augmentation of the edentulous jaw and the single tooth gap.  ***Bone split:*** splitting the alveolar ridge and mobilization of the buccal and oral bone lamella.  ***Bone block:*** transplantation of a bone segment and fixation with screws.  ***Bone substitute material:*** Augmentation with bone substitute material and/or particulate bone (without further procedures)  ***Distraction:*** Mobilisation of a bone segment formed by separation of the jaw by means of a distraction device.  ***Mesh:*** Jaw augmentation with bone substitute material and/or particulate bone held by a titanium mesh. | | Please tick the appropriate value on the ranking scale.  5= is used very frequently  0= is not used by me |
| --- | --- | --- |
| **2.1** | **Related to the athrophic edentulous mandible**  **(interforaminal)** |  |
| 2.1.1 | Bone split | 🞏 🞏 🞏 🞏 🞏 🞏  5 4 3 2 1 0 |
| 2.1.2 | Bone block | 🞏 🞏 🞏 🞏 🞏 🞏  5 4 3 2 1 0 |
| 2.1.3 | Augmentation with bone substitute material and/or particulate bone | 🞏 🞏 🞏 🞏 🞏 🞏  5 4 3 2 1 0 |
| 2.1.4 | Distraction | 🞏 🞏 🞏 🞏 🞏 🞏  5 4 3 2 1 0 |
| 2.1.5 | Mesh | 🞏 🞏 🞏 🞏 🞏 🞏  5 4 3 2 1 0 |
| 2.1.6 | Other | ----------------------------------------------- |
| **2.2** | **Related to the single tooth gap** |  |
| 2.2.1 | Bone split | 🞏 🞏 🞏 🞏 🞏 🞏  5 4 3 2 1 0 |
| 2.2.2 | Bone block | 🞏 🞏 🞏 🞏 🞏 🞏  5 4 3 2 1 0 |
| 2.2.3 | Augmentation with bone substitute material and/or particulate bone | 🞏 🞏 🞏 🞏 🞏 🞏  5 4 3 2 1 0 |
| 2.2.4 | Distraction | 🞏 🞏 🞏 🞏 🞏 🞏  5 4 3 2 1 0 |
| 2.2.5 | Mesh | 🞏 🞏 🞏 🞏 🞏 🞏  5 4 3 2 1 0 |
| 2.2.6 | Other | ----------------------------------------------- |

| **3. Resection**  Do you use resective techniques to achieve a sufficiently wide implant site? (removal of pointed alveolar residual bone) | | Please tick the appropriate value on the ranking scale.  5= is used very frequently  0= is not used by me |
| --- | --- | --- |
| 3.1 | Resection of edentulous alveolar ridge | 🞏 🞏 🞏 🞏 🞏 🞏  5 4 3 2 1 0 |
| 3.2 | Resection in the single tooth gap | 🞏 🞏 🞏 🞏 🞏 🞏  5 4 3 2 1 0 |

|  | | Please tick the appropriate value on the ranking scale.  5= is used very frequently  0= is not used by me |
| --- | --- | --- |
| **4.1** | **Intraoral** |  |
| 4.1.1 | Retromolar region | 🞏 🞏 🞏 🞏 🞏 🞏  5 4 3 2 1 0 |
| 4.1.2 | Mental region | 🞏 🞏 🞏 🞏 🞏 🞏  5 4 3 2 1 0 |
| 4.1.3 | Other | ----------------------------------------------- |
| **4.2** | **Extraoral** |  |
| 4.2.1 | Iliac crest | 🞏 🞏 🞏 🞏 🞏 🞏  5 4 3 2 1 0 |
| 4.2.2 | Cranial calotte | 🞏 🞏 🞏 🞏 🞏 🞏  5 4 3 2 1 0 |
| 4.2.3 | Tibia | 🞏 🞏 🞏 🞏 🞏 🞏  5 4 3 2 1 0 |
| 4.2.4 | Other | ----------------------------------------------- |

| **5. Special implantological diagnostics**  You will be asked about specific diagnostic procedures that you apply in addition to the clinical inspection and X-ray examination. Please estimate: How often do you use this diagnostic procedure.  ***Model analysis:*** measurement of the implant distances, assessment of the width of the alveolar ridge, etc. on the model  ***CBCT:*** Three-dimensional X-ray diagnostics | | Please tick the appropriate value on the ranking scale.  5= is used very frequently  0= is not used by me |
| --- | --- | --- |
| **5.1** | **Related to the athrophic edentulous mandible**  **(interforaminal)** |  |
| 5.1.1 | Model analysis | 🞏 🞏 🞏 🞏 🞏 🞏  5 4 3 2 1 0 |
| 5.1.2 | CBCT | 🞏 🞏 🞏 🞏 🞏 🞏  5 4 3 2 1 0 |
| 5.1.3 | Construction of a drilling template without computer-aided 3D planning | 🞏 🞏 🞏 🞏 🞏 🞏  5 4 3 2 1 0 |
| 5.1.4 | Construction of a drilling template with computer-aided 3D planning | 🞏 🞏 🞏 🞏 🞏 🞏  5 4 3 2 1 0 |
| **5.2** | **Related to the single tooth gap** |  |
| 5.2.1 | Model analysis | 🞏 🞏 🞏 🞏 🞏 🞏  5 4 3 2 1 0 |
| 5.2.2 | CBCT | 🞏 🞏 🞏 🞏 🞏 🞏  5 4 3 2 1 0 |
| 5.2.3 | Construction of a drilling template without computer-aided 3D planning | 🞏 🞏 🞏 🞏 🞏 🞏  5 4 3 2 1 0 |
| 5.2.4 | Construction of a drilling template with computer-aided 3D planning | 🞏 🞏 🞏 🞏 🞏 🞏  5 4 3 2 1 0 |

| **6. Prosthetic Suprastructure**  Please indicate the type of treatment you would prefer for the atrophied edentulous mandible. | | Please tick the appropriate value on the ranking scale.  5= is always  0= is never |
| --- | --- | --- |
| 6.1 | The prosthetic restoration is carried out by the referring dentist. | 🞏 🞏 🞏 🞏 🞏 🞏  5 4 3 2 1 0 |
| 6.2 | A fixed prosthetic restoration is performed. | 🞏 🞏 🞏 🞏 🞏 🞏  5 4 3 2 1 0 |
| 6.3 | A removable prosthetic restoration is performed. | 🞏 🞏 🞏 🞏 🞏 🞏  5 4 3 2 1 0 |

| **Please indicate the approximate extent of your implantological activity?** | |  | |
| --- | --- | --- | --- |
| 8.1 | How many implants have you placed in your practice in the last year?  (Please estimate) | 🞏 0-100  🞏 100-500  🞏 500-1000  🞏 more than 1000 | |
| 8.2 | How many edentulous mandibles did you treat with implants last year? Please estimate the number. | 🞏 0-10  🞏 10-20  🞏 20-50  🞏 more than 50 | |
| 8.3 | Does your practice have X-ray equipment for three-dimensional imaging of the jawbone (CBCT)? | Yes  🞏 | No  🞏 |
